# Discrepancy in Reported and Observed Monoacylglycerol Lipase Inhibition of Aryl Formyl Piperidine Containing Potent and Highly Selective Monoacylglycerol Lipase Inhibitor

**DOI:** 10.1002/cmdc.70525

**Published:** 2026-10-04

**Authors:** Juha R. Savinainen, Praveen Kumar Singh, Jari Yli‐Kauhaluoma, Jayendra Z. Patel

**Affiliations:** ^1^ Institute of Biomedicine, School of Medicine, Faculty of Health Sciences University of Eastern Finland Kuopio Finland; ^2^ Drug Research Program, Division of Pharmaceutical Chemistry and Technology, Faculty of Pharmacy University of Helsinki Helsinki Finland

**Keywords:** 2‐arachidonoyl glycerol, inhibitors, monoacylglycerol lipase, selective

## Abstract

Zhi et al. reported compound **36** as a potent reversible inhibitor of human recombinant monoacylglycerol lipase (hrMAGL; IC_50_ = 15 nM) with excellent selectivity over other targets. To develop potential PET tracers for imaging MAGL, we synthesized compound **36** as a reference compound. Surprisingly, we observed a significant discrepancy between the reported potency and our findings in two well‐established in vitro assays. Compound **36** exhibited no inhibition of endocannabinoid hydrolysis in HEK293 lysates overexpressing human MAGL in a glycerol‐based assay at 1 μM. In the competitive ABPP assay, it appeared completely inactive inhibiting MAGL in mouse whole‐brain proteome even at 100 μM. In addition, eight structural analogs (**2**–**9**) of compound **36** also failed to show any significant MAGL inhibition. Interestingly, when the synthetic substrate 4‐nitrophenyl acetate, also used by Zhi et al., was employed, compound **36** inhibited MAGL more effectively than in glycerol‐based assay, with an IC_50_ value of 143 nM, being almost 10‐fold less potent than reported. In our reversibility assay, compound **36** exhibited measurable MAGL inhibition only at a concentration of 100 μM. In summary, this study contradicts the earlier report and therefore, compound **36** should be used with caution as a research tool in studies exploring MAGL inhibition.

## Graphical abstract

A reported potent and selective reversible MAGL inhibitor fails to reproduce its claimed potency in two established in vitro assays, even when the previously reported substrate is used, while eight related analogs are likewise inactive. The findings highlight substantial assay‐dependent behavior and the need for careful validation before such compounds are employed as MAGL research tools.

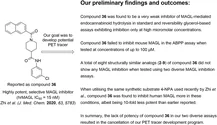

## 1 Introduction

Monoacylglycerol lipase (MAGL), a 33‐kDa membrane‐associated enzyme, is a member of the serine hydrolase superfamily responsible for the hydrolysis of monoacylglycerols (MAGs) in the body [1, 2]. In the central nervous system (CNS), 2‐arachidonoylglycerol (2‐AG)—the primary endocannabinoid—is degraded into arachidonic acid and glycerol primarily by MAGL, accounting for approximately 85% of 2‐AG hydrolysis [2]. Three other serine hydrolases, *α*/*β*‐hydrolase domain‐containing 6 (ABHD6), *α*/*β*‐hydrolase domain‐containing 12 (ABHD12), and fatty acid amide hydrolase (FAAH), contribute less, accounting for approximately 4%, 9%, and 1% of 2‐AG hydrolysis, respectively [3, 4, 5]. By regulating the levels of endocannabinoids, MAGL plays a pivotal role in diverse physiological processes, including pain modulation, appetite regulation, memory, mood stabilization, and inflammation [6, 7, 8, 9]. In addition, MAGL has been reported to be involved in the regulation of the fatty acid network that promotes cancer pathogenesis [10]. Inhibition of MAGL can lead to increased levels of 2‐AG, thereby influencing endocannabinoid signaling in the body. Modulating the functions of MAGL has garnered significant attention as a potential target for therapeutic interventions in various fields, including pain management, inflammation, neurodegenerative disorders, cancers, and other conditions related to the endocannabinoid system [11, 12, 13].

Over the last 18 years, various irreversible and reversible MAGL inhibitors targeting either the nucleophilic Ser^122^ residue of the catalytic triad (Ser^122^–Asp^239^–His^269^) present in the active site of MAGL or cysteine residues (Cys^201^, Cys^208^, and Cys^242^) located near the catalytic site of MAGL have been reported [11, 12, 13]. Among these, urea [14, 15, 16, 17, 18, 19], carbamate [20, 21, 22, 23, 24, 25], and thiocarbamate [26] functionality‐containing compounds are well‐characterized as highly potent and selective MAGL inhibitors (Figure 1A). The majority of these are irreversible MAGL inhibitors, except CK16, MJN110, and JZP‐361, which are documented as reversible inhibitors. Several compounds containing amide functionalities (Figure 1B) are also well‐characterized as highly potent and selective MAGL inhibitors, exhibiting a reversible mode of inhibition toward MAGL [27, 28, 29, 30, 31, 32, 33, 34, 35, 36, 37, 38]. Reversible MAGL inhibitors are considered safer alternatives to irreversible ones, which are associated with adverse effects such as physical dependance and CB1 receptor desensitization in the brain, arising from sustained receptor activation due to permanent MAGL blockade [39, 40]. Among the reported reversible MAGL inhibitors, compound **36** (Figure 1B), reported by Zhi et al. [32], features a bis‐amide functionality and is perhaps the only one that can be synthesized effortlessly in two synthetic steps. Moreover, this compound has been reported as a highly potent and reversible inhibitor of human recombinant MAGL (hrMAGL, IC_50_ = 15 nM) with high selectivity toward MAGL over potential off‐targets in the endocannabinoid system, namely, FAAH, CB1, and CB2 [32]. In short, considering its ease of synthesis, low nanomolar potency, and reversible mode of action toward MAGL, we considered compound **36** as a reference compound and aimed to develop potential precursors, which can be utilized for the development of a potential positron emission tomography (PET) tracer for imaging MAGL.

**FIGURE 1:**
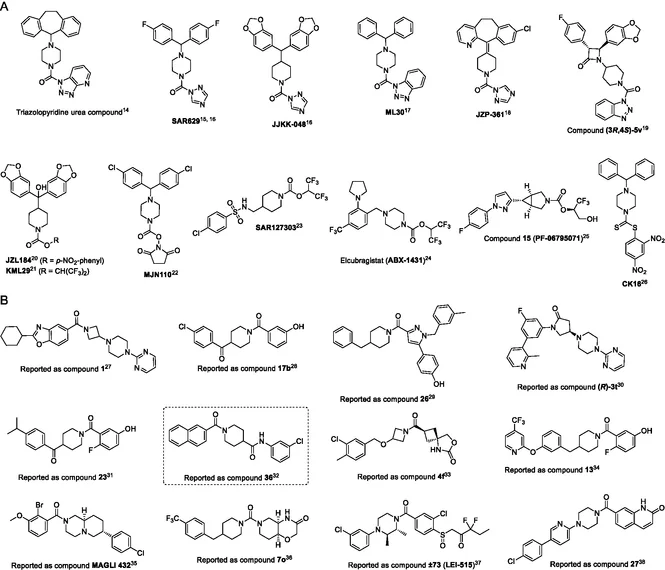
(A) Urea, carbamate, and thiocarbamate series of compounds as highly potent and selective MAGL inhibitors. (B) Amide‐containing (except compound **7o**, which is a tetrasubstituted urea) highly potent and selective reversible MAGL inhibitors (Note: compounds numbering or codes are as per the original reported articles).

PET is a noninvasive and highly sensitive technology that allows for the quantification of biochemical and pharmacological processes in vivo under normal and disease conditions without exhibiting pharmacological action or disturbing the biological state [41, 42, 43]. In this regard, we aim to conduct PET studies of MAGL to gain an in‐depth understanding of MAGL‐related pathological changes between normal and disease states, as well as the in vivo interactions of novel MAGL inhibitors with the target [44]. To date, our group and others have disclosed a series of MAGL PET tracers, including both irreversible [45, 46, 47, 48, 49, 50, 51] and reversible [49, 52, 53, 54, 55, 56, 57, 58]. As noted by Liang et al., reversible MAGL PET tracers offer several advantages over irreversible tracers [55]. Consequently, we aimed to develop a reversible MAGL PET tracer based on compound **36**. Therefore, we synthesized compound **36** and to confirm its high potency toward MAGL, we used our well‐established glycerol‐based assay for human MAGL. In addition, to validating its reported selectivity toward MAGL over other endocannabinoid targets, particularly FAAH, and ABHD6, we conducted an activity‐based protein profiling (ABPP) assay using a mouse whole‐brain proteome. Based on the results of these two assays, we synthesized a small set of structurally related analogs and present our findings herein.

## 2 Results and Discussion

### 2.1 Chemistry

A convenient synthesis of compound **36** and its analogs **2**–**9** was performed according to the previously reported methods (Scheme 1 and Figure 2) [32]. Commercially available 1‐(*tert*‐butoxycarbonyl)piperidine‐4‐carboxylic acid **10** was coupled with an appropriate aniline **11** using hexafluorophosphate azabenzotriazole tetramethyl uronium (HATU) as a coupling agent to give Boc‐containing amide analog **12**. The removal of Boc protecting group of amide **12** was carried out using TFA to yield amine **13**, which was then coupled with the appropriate carboxylic acid **14** using HATU to afford targeted compounds **36** and **2**–**9**.

**SCHEME 1:**
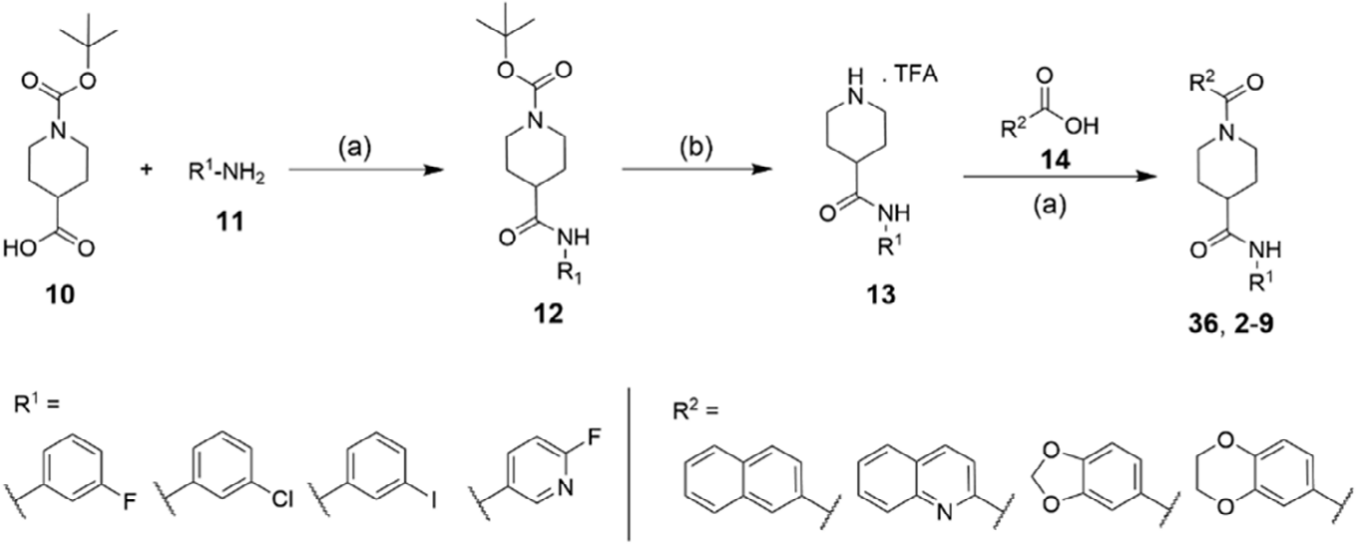
Synthesis of compounds **36** and **2**–**9**. Reagents and conditions: (a) HATU, DIPEA, DCM, 25 °C, 16 h and (b) TFA, DCM, 0–25 °C, 16 h.

**FIGURE 2:**
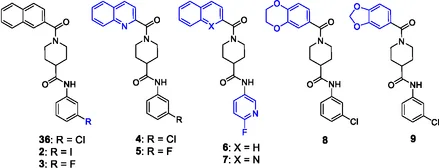
Chemical structures of compounds **36** and **2**–**9** (the blue color highlights the main difference between that particular compound and compound **36**).

### 2.2 Determination of MAGL and ABHD6 Activities by a Sensitive Fluorescent Glycerol Assay Utilizing Natural Substrate 1‐AG

To compare basic inhibitor activity against control endocannabinoid hydrolase inhibitors, the activities of the synthesized compounds were tested at 1 μM screening concentration against human MAGL in overexpressing HEK293 cell lysates (Figure 3A), as per our previously reported method [16, 18, 59]. Unexpectedly, compound **36** was found to be inactive toward human MAGL at a concentration of 1 μM, even though it was reported as highly potent. Similarly, eight structural analogs (compounds **2**–**9**) of compound **36** were also found to be inactive at this concentration. It should be noted that reference compound JJKK‐048 behaved as anticipated by effectively inhibiting MAGL enzyme [16].

**FIGURE 3:**
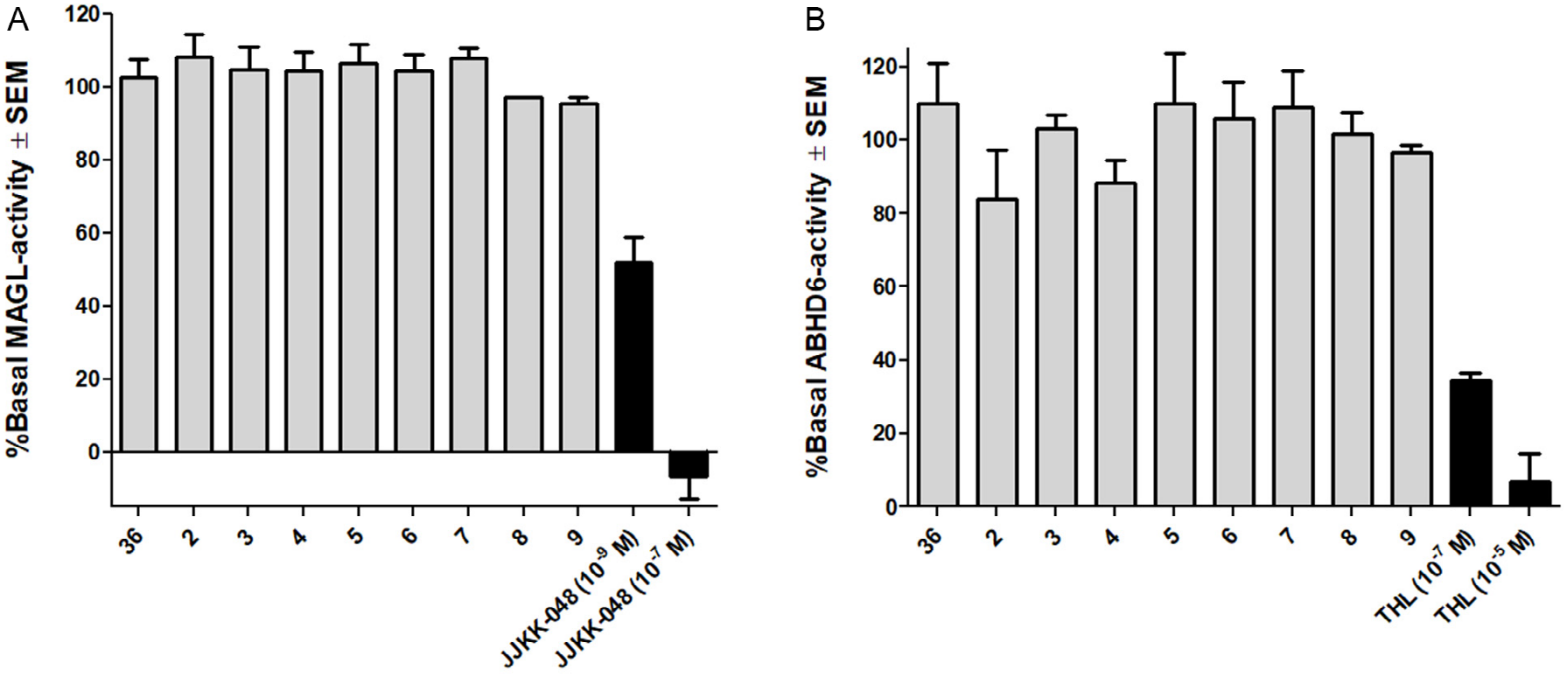
Inhibition activity of compounds **36** and **2**–**9** at 1 µM concentration in HEK293 cell lysates overexpressing human MAGL (A) and human ABHD6 (B). The compounds were tested in glycerol‐based enzymatic assay, as described in the Methods section. JJKK‐048 was used as a reference MAGL [16] inhibitor while THL was used as a reference ABHD6 inhibitor [59]. The data are from 2 to 4 independent experiments performed in duplicates (average ± SEM).

In short, replacing the *meta*‐chloro substituent in compound **36** with either *meta*‐iodo (compound **2**) or *meta*‐fluoro (compound **3**) did not affect MAGL inhibition. Similarly, replacing the bicyclic naphthyl ring in compound **36** with other bicyclic rings—quinoline (compound **4**), 2,3‐dihydrobenzo[*b*][1,4]dioxine (compound **8**), or benzo[*d*][1,3]dioxole (compound **9**)—also resulted in a lack of MAGL inhibition. Furthermore, replacing the *meta*‐chloro substituent in the quinoline‐containing compound **4** with *meta*‐fluoro (compound **5**) did not improve MAGL inhibition. In addition, the naphthyl ring‐containing compound **6** and the quinoline‐containing compound **7**, both bearing a 6‐fluoro‐substituted pyridine ring, also showed no MAGL inhibitory activity. In short, none of the synthesized analogs, including compound **36**, which was claimed to be a highly potent and selective MAGL inhibitor, did not exhibit any detectable MAGL inhibition. It should be noted that our analytical data for compound **36** is consistent with earlier reports [32], and compounds **2**–**9** are structurally close to analogs of compound **36**. Next, the inhibitory activities of compounds **36** and **2**–**9** were tested at a concentration of 1 μM against human ABHD6 (Figure 3B) using our previously validated method [60, 61]. Herein, none of the compounds showed any significant inhibition (≤20%) toward human ABHD6. Overall, all synthesized compounds failed to show any noticeable inhibition towards both human MAGL and ABHD6.

### 2.3 ABPP Using Mouse Brain Membrane Proteome

As none of the synthesized compounds **36** and **2**–**9** exhibited any stronger impact on MAGL inhibition in our kinetic enzymatic assay, we wanted to confirm these unexpected findings by using another in vitro approach, a competitive ABPP assay. This assay has been extensively used by us and others to cross‐check the compounds‐selectivity to inhibit MAGL over other endocannabinoid hydrolases, such as ABHD6, ABHD12, and FAAH [18, 20, 21, 22, 24, 25, 60, 61, 62]. For this purpose, we tested compounds **36** and **2**–**9** at two different concentrations (1 and 10 µM) using competitive ABPP of the mouse brain membrane proteome (Figure 4A). We used our previously reported inhibitors JJKK‐048 [16], JZP‐430 [60], and JZP‐327A [62] at the indicated concentrations to locate the bands of MAGL, ABHD6, and FAAH, respectively. In two separate assay settings, each repeated twice, compounds **36** and **2**–**9** failed to inhibit the MAGL band at both concentrations. Indeed, as shown in Figure 4B, compound **36** failed to inhibit mouse MAGL even at 100 µM concentration. However, only when tested at a very high concentration (400 µM), it showed some MAGL inhibition, albeit not complete, as well as inhibition of ABHD6 and FAAH. It should be noted that all reference compounds behaved as expected by successfully inhibiting their respective bands.

**FIGURE 4:**
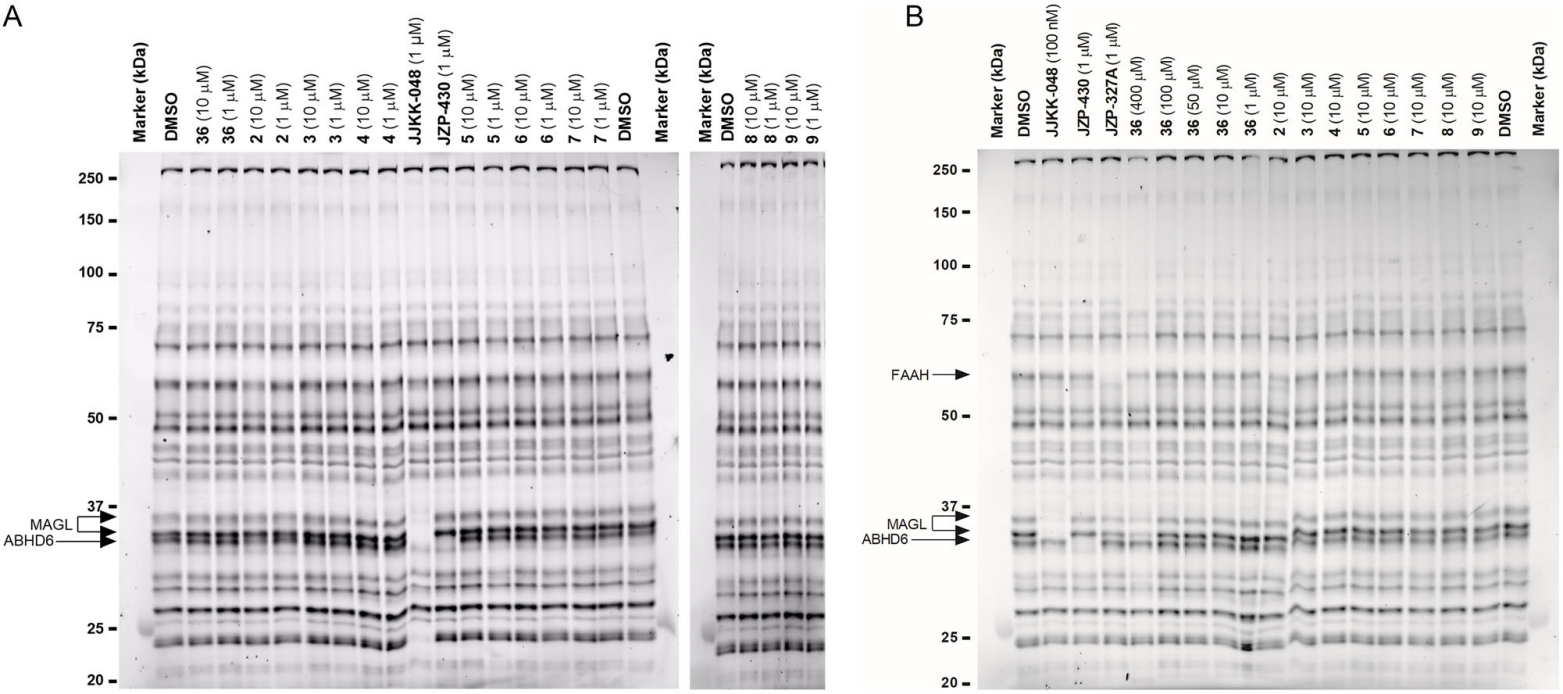
Competitive ABPP of compounds **36** and **2**–**9** among the serine hydrolases in the mouse whole‐brain membrane proteome. Molecular weight markers are indicated on the left. Reference inhibitors JJKK‐048, JZP‐430, and JZP‐327A were used at the indicated concentrations to identify the following serine hydrolases from the gel: MAGL (inhibited by JJKK‐048) [16], ABHD6 (inhibited by JZP‐430) [60] and FAAH (inhibited by JZP‐327A) [62]. Additionally, protein bands corresponding to MAGL (doublet), ABHD6, and FAAH are indicated. Note that JJKK‐048 at higher concentration (1 µM, Figure 4A) starts to inhibit several other serine hydrolases than MAGL, but is selective in routinely used concentration 100 nM (Figure 4B). Note also that compound **36**, as well as the other compounds, failed to inhibit probe labeling of MAGL at test concentrations of 1 and 10 µM. Compound **36** also failed to inhibit probe labeling of MAGL at test concentrations of 100 µM while only at a test concentration of 400 µM did show some inhibition of MAGL, along with inhibition of ABHD6 and FAAH. The gel is representative of two ABPP experiments with similar outcomes.

### 2.4 Determination of MAGL Activity Using 4‐Nitrophenyl Acetate as a Substrate

To further investigate why compound **36** appeared to be a very weak inhibitor of both human and mouse MAGL in our glycerol assay (that is based on hydrolysis of the natural endocannabinoid 1‐AG), and in ABPP assay, we next evaluated its ability to inhibit MAGL‐mediated hydrolysis of the chromogenic esterase substrate 4‐nitrophenyl acetate (4‐NPA). This synthetic substrate was utilized by Zhi et al. [32] and may thus have a potential impact on their contradicting conclusion.

In our hands, compound **36** was indeed capable of inhibiting human MAGL‐mediated 4‐NPA (100 μM) hydrolysis, much more effectively than in glycerol‐based assay, yielding an IC_50_ value of 143 nM (logIC_50_ = −6.85 ± 0.08) (Figure 5). Notable is, however, that this value is still approximately 10‐fold less potent than that reported by Zhi et al. for recombinant MAGL (IC_50_ = 15 nM) [32]. In contrast, our reference MAGL inhibitor JJKK‐048 inhibited 4‐NPA hydrolysis with an IC_50_ of 164 pM (logIC_50_ = −9.79 ± 0.14), which is comparable to the potency previously observed in our glycerol assays using the natural endocannabinoid substrate (logIC_50_ = −9.44 ± 0.05) [16]. These findings suggest that JJKK‐048 inhibits both 1‐AG and 4‐NPA hydrolysis with similar efficacy, whereas compound **36** is markedly less effective at inhibiting hydrolysis of the natural MAGL substrate 1‐AG than that of the artificial esterase substrate 4‐NPA.

**FIGURE 5:**
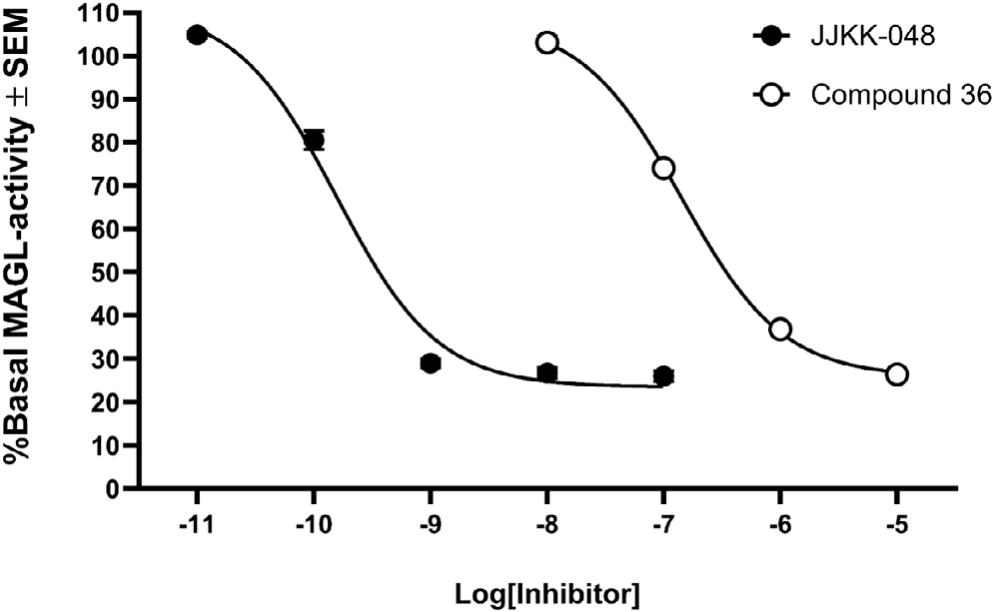
MAGL assay format utilizing hydrolysis of chromogenic esterase substrate 4‐NPA. The results suggest that compound **36** is more active in inhibiting human MAGL with artificial substrate 4‐NPA than with 1‐AG, a natural MAGL substrate used in glycerol assays. Dose‐dependent inhibition of 3HA‐hMAGL was analyzed in comparison to the reference MAGL inhibitor JJKK‐048 [16]. Hydrolysis of 4‐NPA to 4‐nitrophenol was measured at absorption maximum of 405 nm as described in the Methods. The results are from at least 3 independent experiments performed in duplicates (average ± SEM).

One possible explanation to the discrepant results between inhibition of 4‐NPA and 1‐AG hydrolysis for compound **36** is that 1‐AG is a larger and more lipophilic molecule, and its endogenous binding site within MAGL may therefore be more hydrophobic and structurally preferable than that utilized by the smaller and more water‐soluble substrate 4‐NPA. On the other hand, the active‐site‐directed fluorophosphonate‐probe TAMRA‐FP used in our ABPP experiments, which reportedly binds within the catalytic pocket of MAGL, neither was displaced by compound **36** even at high 100 μM concentration, further suggesting very low binding potency for compound **36**. This finding supports the hypothesis that compound **36** may not bind effectively to the MAGL active site and therefore would not be suitable inhibitor in inhibiting endocannabinoid hydrolysis in vitro and therefore in increasing endocannabinoid levels in vivo studies either.

### 2.5 Reversibility Study for MAGL‐Inhibitors Using Natural Substrate 1‐AG

Zhi et al. also claimed in their previous report that compound **36** acts as a reversible MAGL inhibitor [32]. If true, this may potentially explain our observation of very weak inhibitor activity in the used assays. To explore this possibility, the reversibility of MAGL inhibition was evaluated using an assay format previously described by us [18]. Compound **36** and tested reversible reference compounds (JNJ‐42226 314 [63] and ATM‐113 [18, 64]) as well as irreversible inhibitor JJKK‐048 [16] were tested using a dilution‐based protocol utilizing HEK293 cell lysates expressing 3 HA‐hMAGL, as described in detail in the methods.

As a result, compound **36** displayed very weak inhibition also in the reversibility assay. It inhibited hMAGL only partially (23 ± 2%, SEM, *n* = 6) at a very high concentration of 100 µM, while remaining inactive at 10 µM (Figure 6). At 100 µM, however, the inhibitory effect appeared to diminish as the reaction progressed beyond 45 min (up to the final 90 min), suggesting recovery of enzyme activity (reversibility) over time. The reference MAGL inhibitors JNJ‐42226314 [63] and AMT‐113 [18, 64] both showed the expected reversible binding mode, whereas JJKK‐048 [16] behaved as an irreversible inhibitor (Figure 6). Taken together, despite its ability to compete modestly with the artificial MAGL substrate 4‐NPA (Figure 6), these findings suggest that compound **36** acts only as a very weak inhibitor of MAGL‐mediated endocannabinoid hydrolysis.

**FIGURE 6:**
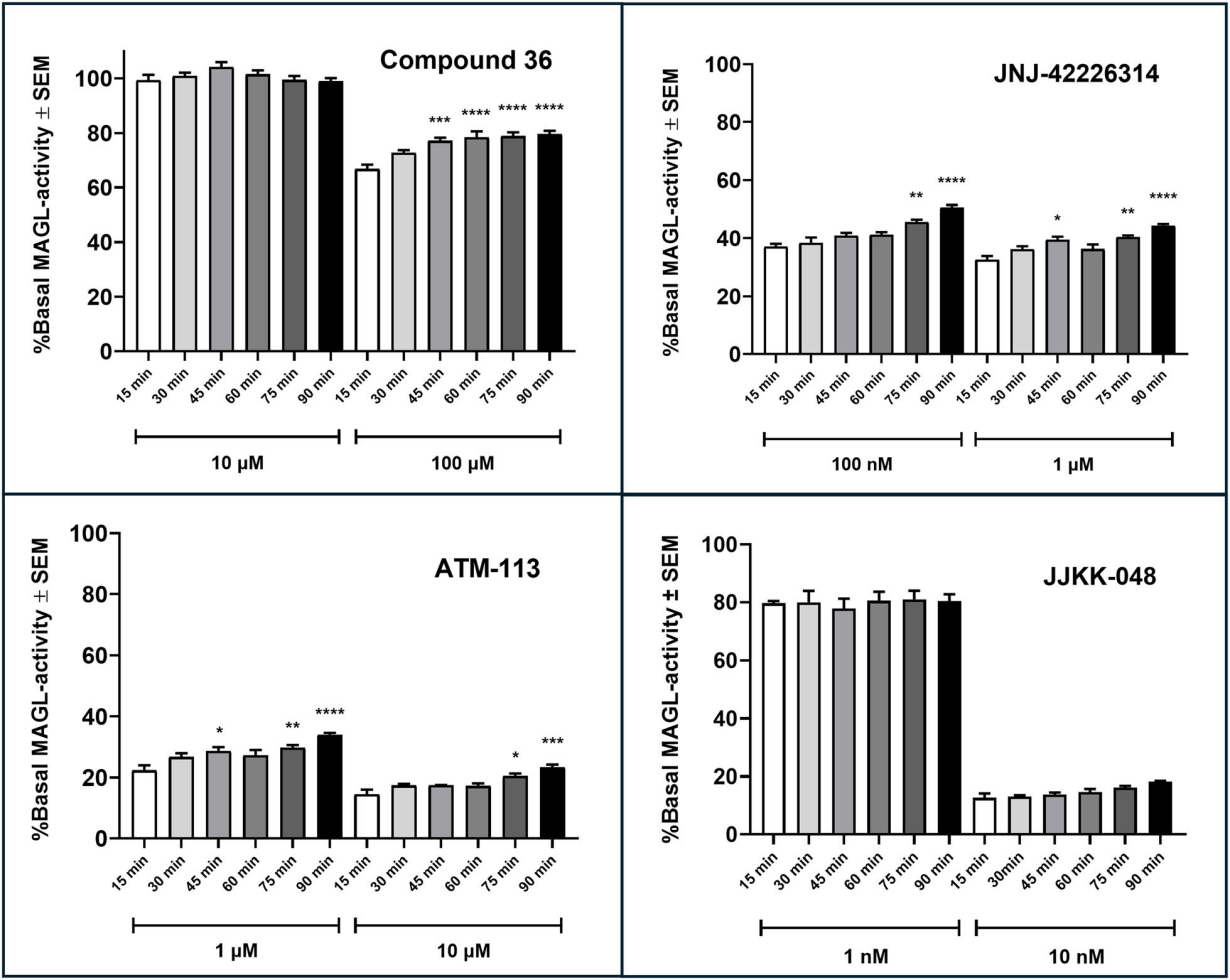
Reversibility assay monitoring the hydrolysis of the endogenous MAGL substrate 1‐AG to glycerol by human MAGL confirmed the weak inhibitory potency of compound **36** and simultaneously revealed reversible inhibition at the high concentration of 100 μM. Previously reported reversible MAGL inhibitors JNJ‐42226314 [63] and ATM‐113 [18, 64] showed significantly reduced inhibition over the course of the incubation period (compared with the 15 min time point), whereas the MAGL inhibitor JJKK‐048 [16] displayed an irreversible binding mode with no significant changes in inhibitory activity. hMAGL‐mediated hydrolysis of 1‐AG was analyzed following a 60 min preincubation with two inhibitor concentrations. The enzyme‐inhibitor mixtures were then rapidly diluted 40‐fold with substrate solution, and product formation was monitored immediately by kinetic fluorescence measurement, as described in the Methods. Data represent three independent experiments performed in duplicate and are presented as mean ± SEM. Statistical comparisons of individual time points versus the corresponding 15 min time point at each inhibitor concentration were performed using one‐way ANOVA followed by Tukey's multiple‐comparisons test (*p < 0.05, **p < 0.01, ***p < 0.001, and ****p < 0.0001).

## 3 Conclusion

In summary, compound **36**, which was previously reported as a highly potent and selective MAGL inhibitor, and along with its eight structurally related compounds synthesized herein, failed to demonstrate significant MAGL inhibition in either of our two well‐established distinct assays employing the natural endocannabinoid 1‐AG (glycerol‐based assays) or TAMRA‐FP probe (ABPP assay), both of compounds which bind within the catalytic cavity of MAGL. Also, in the presence of the synthetic substrate 4‐NPA, compound **36** was found to be approximately 10‐fold less potent at inhibiting MAGL than the earlier reported IC_50_ value. Furthermore, unlike the reference MAGL inhibitors, compound **36** exhibited only a very weak inhibition of MAGL‐mediated endocannabinoid hydrolysis in reversibility experiments, remaining inactive at high micromolar concentrations. Due to this significant discrepancy between the reported potency of compound **36** and our findings regarding its lack of inhibition of MAGL in two diverse assays, we have halted our PET tracer development program aimed at imaging MAGL. Therefore, we recommend that the use of this compound as a research tool in future biological and pharmacological studies exploring MAGL inhibition be approached with caution.

## 4 Experimental Protocols

### 4.1 Chemistry

The materials (reagents, solvents, reference compounds, and chemicals) were purchased from selected vendors such as Merck (Espoo, Finland), Fluorochem (Hadfield, UK), A2B Chem LLC (San Diego, USA), Ambeed (Arlington Heights, Illinois, US), Thermo Fisher Scientific Oy (Vantaa, Finland), and VWR (Helsinki, Finland), and used further without purification. All reactions were carried out under an inert argon or nitrogen atmosphere using oven‐dried glassware and dry solvents. The progress of the reaction was monitored by thin‐layer chromatography (TLC) using aluminum plates coated with 0.2 mm Silica gel 60 F_254_ (Merck, Espoo, Finland) and solvent system of either 80% ethyl acetate (EtOAc) in heptane or 5–15% methanol (MeOH) in dichloromethane (DCM) and finally visualizing TLC through UV light (254/366 nm). Flash chromatography was used to carry out for the purification of synthesized compounds using Biotage Isolera Spektra System with ACI and Assist (ISO‐1SW Isolera One), equipped with a variable UV−VIS (200 − 800 nm) photodiode array (Uppsala, Sweden). Herein, prepacked Biotage SNAP and Sfär SiO_2_ columns, along with samplet, were used as the stationary phase, while solvent combinations as mentioned in general synthetic methods were used as the mobile phase. For the characterization of compounds, nuclear magnetic resonance spectra (^1^H, ^13^C, and ^19^F NMR) were recorded on a Bruker Ascend 400 MHz—Avance III HD NMR spectrometer (Bruker Corporation, Billerica, MA, USA) using DMSO‐*d*_6_. For processing the recorded FID files, MestReNova 14.1.0 software (Mestrelab Research, Santiago de Compostela, Spain) was used. Chemical shifts (*δ*) are quoted in parts per million (ppm) using residual solvent peaks DMSO‐*d*_6_ 2.50 and 39.52 ppm for ^1^H and ^13^C NMR, respectively as standards. The multiplicity of each signal is represented using the following abbreviations: s (singlet), d (doublet), t (triplet), br s (broad singlet), dd (doublet of doublets), ddd (doublet of doublet of doublets), dt (doublet of triplets), td (triplet of doublet), tt (triplet of triplets), and m (multiplet). Coupling constants *J* are reported in hertz (Hz). The accurate mass and purity (>95%) of all synthesized compounds were confirmed by in‐house liquid chromatography−mass spectrometry (LC−MS) analyses with a Waters Acquity1 UPLC system (Waters, Milford, MA) equipped with an Acquity1 UPLC BEH C18 column (1.7 μm, 50 × 2.1 mm, Waters, Drinagh, Ireland), an Acquity PDA detector, and a Waters Synapt G2 HDMS mass spectrometer (Waters, Milford, Massachusetts, US) via an electrospray ionization (ESI) ion source in a positive mode. High‐resolution mass (HRMS‐ESI) data is reported for the molecular ions [M + H]^+^ and measured for compounds **36** and **2**–**9**.

### 4.2 General Procedure for the Synthesis of Compounds **36** and **2**–**9**

The previously reported method was modified as follows [32]. A mixture of *N*‐Boc‐piperidine‐4‐carboxylic acid (**10**) (4.4 mmol), DIPEA (6.5 mmol), and HATU (5.2 mmol) in DCM (45 mL) was stirred at room temperature for 30 min. Then, appropriately substituted amine (**11**) (4.4 mmol) was added to the reaction mixture, which was stirred at room temperature for 6 h. The reaction mixture was washed sequentially with water and 1 M hydrochloric acid and/or a 10% solution of sodium bicarbonate in water (note: for quinoline and pyridine‐containing analogs hydrochloric acid wash was omitted), then dried over anhydrous sodium sulfate. The solvent was removed under reduced pressure to obtain the crude Boc‐containing amide analog (**12**), which was used without further purification and characterization.

Next, 2 mL of TFA was added to a solution of the crude Boc‐containing amide analog (**12**) in DCM (45 mL) at 0–5 °C, and the mixture was stirred at room temperature for 16 h. After removing the solvents under vacuum, DMF (10 mL) was added to the trifluoroacetate salt of amine (**13**) (2.2 mmol). To this solution, DIPEA (8.90 mmol) was added at 0–5 °C, and the resulting mixture was stirred at room temperature for 30 min. Next, the appropriate acid compound (**14**) (2.91 mmol) and HATU (3.41 mmol) were added, and the resulting mixture was stirred at room temperature for 16 h. The mixture was diluted with water and extracted with EtOAc. The organic layer was washed sequentially five times with water, followed by saturated brine, and finally dried over anhydrous Na_2_SO_4_. The organic solvent was removed under vacuum using a rotary evaporator, and the crude compound was purified by column chromatography on silica gel via elution with either 70–80% EtOAc in heptane or 3–15% MeOH in DCM to give the desired compounds **36** and **2**–**9**.

### 4.3 1‐(2‐Naphthoyl)‐*N*‐(3‐chlorophenyl)piperidine‐4‐carboxamide (**36**)

The above‐mentioned general procedure was followed using *N*‐Boc‐piperidine‐4‐carboxylic acid **10** (1.03 g, 4.41 mmol), 3‐chloroaniline (0.556 g, 4.41 mmol), and 2‐naphthalenecarboxylic acid (0.500 g, 2.93 mmol). Compound **36** was obtained as white solid (0.367 g, 46%); ^1^H‐NMR (400 MHz, DMSO‐*d*_6_): *δ* 10.13 (s, 1H), 8.07–7.93 (m, 4H), 7.84 (t, *J* = 2.1 Hz, 1H), 7.63–7.55 (m, 2H), 7.55–7.43 (m, 2H), 7.33 (t, *J* = 8.1 Hz, 1H), 7.09 (ddd, *J* = 8.0, 2.2, 1.0 Hz, 1H), 4.57–4.51 (br s, 1H), 3.73–3.66 (br s, 1H), 3.15–2.84 (br s, 2H), 2.65 (tt, *J* = 11.3, 3.3 Hz, 1H), 1.99–1.52 (m, 4H); ^13^C NMR (101 MHz, DMSO‐*d*_6_) *δ* 173.2, 169.1, 140.7, 133.6, 133.0, 133.0, 132.3, 130.4, 128.3, 128.1, 127.7, 127.0, 126.7, 126.1, 124.3, 122.8, 118.6, 117.5, 46.0, 42.7, 40.9, 28.7, 28.0; HRMS‐ESI (*m/z*): calcd for C_23_H_22_ClN_2_O_2_ [M + H]^+^ 393.1370, found: 393.1382.

### 4.4 1‐(2‐Naphthoyl)‐*N*‐(3‐iodophenyl)piperidine‐4‐carboxamide (**2**)

The above‐mentioned general procedure was followed using *N*‐Boc‐piperidine‐4‐carboxylic acid **10** (1.02 g, 4.43 mmol), 3‐iodoaniline (0.955 g, 4.43 mmol), and 2‐naphthalenecarboxylic acid (0.996 g, 5.83 mmol). Compound **2** was obtained as white solid (1.31 g, 61%); ^1^H‐NMR (400 MHz, DMSO‐*d*_6_): *δ* 10.04 (s, 1H), 8.13 (t, *J* = 1.9 Hz, 1H), 8.08–7.91 (m, 4H), 7.65–7.47 (m, 4H), 7.39 (dt, *J* = 7.8, 1.3 Hz, 1H), 7.09 (t, *J* = 8.0 Hz, 1H), 4.56 (s, 1H), 3.73 (s, 1H), 2.93 (s, 2H), 2.64 (tt, *J* = 11.4, 3.7 Hz, 1H), 2.08–1.42 (m, 4H); ^13^C NMR (101 MHz, DMSO‐*d*_6_) *δ* 173.1, 169.1, 140.6, 133.6, 133.0, 132.3, 131.6, 130.7, 128.3, 128.1, 127.7, 127.3, 127.0, 126.7, 126.1, 124.3, 118.3, 94.5, 46.8, 42.7 (2C), 29.2, 28.2; HRMS‐ESI (*m/z*): calcd for C_23_H_22_IN_2_O_2_ [M + H]^+^ 485.0726, found: 485.0735.

### 4.5 1‐(2‐Naphthoyl)‐*N*‐(3‐fluorophenyl)piperidine‐4‐carboxamide (**3**)

The above‐mentioned general procedure was followed using *N*‐Boc‐piperidine‐4‐carboxylic acid **10** (1.03 g, 4.43 mmol), 3‐fluoroaniline (0.533 g, 4.43 mmol), and 2‐naphthalenecarboxylic acid (0.982 g, 5.82 mmol). Compound **3** was obtained as a white solid (0.20 g, 13%); ^1^H‐NMR (400 MHz, DMSO‐*d*_6_): *δ* 10.16 (s, 1H), 8.00 (dt, *J* = 12.3, 7.5 Hz, 4H), 7.69–7.45 (m, 4H), 7.33 (t, *J* = 5.8 Hz, 2H), 6.90–6.82 (m, 1H), 4.58 (s, 1H), 3.74 (s, 1H), 3.22–2.80 (m, 2H), 2.74–2.58 (m, 1H), 1.91–1.62 (m, 4H); ^19^F NMR (376 MHz, DMSO‐*d*_6_) *δ* −112.10; ^13^C NMR (101 MHz, DMSO‐*d*_6_) *δ* 173.7, 169.5, 163.8, 141.5, 141.4, 134.1, 133.5, 132.8, 130.8, 130.7, 128.8, 128.6, 128.2, 127.5, 127.2, 126.6, 124.8, 115.3, 110.1, 109.9, 106.5, 106.2, 43.2; HRMS‐ESI (*m/z*): calcd for C_23_H_22_FN_2_O_2_ [M + H]^+^ 377.1665, found: 377.1682.

### 4.6 *N*‐(3‐Chlorophenyl)‐1‐(quinoline‐2‐carbonyl)piperidine‐4‐carboxamide (**4**)

The above‐mentioned general procedure was followed using *N*‐Boc‐piperidine‐4‐carboxylic acid **10** (1.03 g, 4.42 mmol), 3‐chloroaniline (0.556 g, 4.42 mmol), and 2‐quinolinecarboxylic acid (0.315 g, 1.82 mmol). Compound **4** was obtained as white solid (0.410 g, 51%); ^1^H‐NMR (400 MHz, DMSO‐*d*_6_): *δ* 10.14 (s, 1H), 8.50 (dd, *J* = 8.6, 0.8 Hz, 1H), 8.09–8.00 (m, 2H), 7.88–7.79 (m, 2H), 7.72–7.64 (m, 2H), 7.47 (ddd, *J* = 8.3, 2.0, 1.0 Hz, 1H), 7.32 (t, *J* = 8.1 Hz, 1H), 7.08 (ddd, *J* = 8.0, 2.1, 1.0 Hz, 1H), 4.67–4.54 (m, 1H), 3.86–3.74 (m, 1H), 3.16 (ddd, *J* = 13.4, 11.7, 3.2 Hz, 1H), 2.96 (td, *J* = 12.7, 3.0 Hz, 1H), 2.75–2.62 (m, 1H), 1.97 (dd, *J* = 13.2, 3.8 Hz, 1H), 1.83–1.62 (m, 3H); ^13^C NMR (101 MHz, DMSO‐*d*_6_) *δ* 173.2, 166.7, 154.4, 146.1, 140.7, 137.4, 133.0, 130.4, 130.3, 129.0, 128.0, 127.6, 127.5, 122.8, 120.2, 118.6, 117.5, 46.0, 42.7, 40.9, 28.7, 28.0; HRMS‐ESI (*m/z*): calcd for C_22_H_21_ClN_3_O_2_ [M + H]^+^ 394.1322, found: 394.1336.

### 4.7 *N*‐(3‐Fluorophenyl)‐1‐(quinoline‐2‐carbonyl)piperidine‐4‐carboxamide (**5**)

The above‐mentioned general procedure was followed using *N*‐Boc‐piperidine‐4‐carboxylic acid **10** (1.01 g, 4.41 mmol), 3‐fluoroaniline (0.533 g, 4.41 mmol), and quinoline‐2‐carboxylic acid (0.987 g, 5.82 mmol). Compound **5** was obtained as a white solid (0.60 g, 36%); ^1^H‐NMR (400 MHz, DMSO‐*d*_6_): *δ* 10.18 (s, 1H), 8.51 (d, *J* = 8.5 Hz, 1H), 8.05 (dd, *J* = 8.0, 1.9 Hz, 2H), 7.85–7.81 (m, 1H), 7.74–7.58 (m, 3H), 7.36–7.32 (m, 2H), 6.89–6.84 (m, 1H), 4.61 (dd, *J* = 10.8, 6.7 Hz, 1H), 3.81 (dd, *J* = 13.5, 4.1 Hz, 1H), 3.20–3.16 (m, 1H), 3.01–2.93 (m, 1H), 1.98 (dd, *J* = 13.3, 3.5 Hz, 1H), 1.84–1.63 (m, 3H); ^19^F NMR (376 MHz, DMSO‐*d*_6_) *δ* −112.11; ^13^C NMR (101 MHz, DMSO‐*d*_6_) *δ* 173.7, 167.2, 154.9, 146.6, 141.5, 141.4, 137.9, 130.8, 129.5, 128.5, 127.9, 120.6, 115.3, 110.1, 109.9, 106.5, 106.3, 46.5, 43.2, 41.4, 38.7, 28.5; HRMS‐ESI (*m/z*): calcd for C_22_H_21_FN_3_O_2_ [M + H]^+^ 378.1618, found: 378.1642.

### 4.8 1‐(2‐Naphthoyl)‐*N*‐(6‐fluoropyridin‐3‐yl)piperidine‐4‐carboxamide (**6**)

The above‐mentioned general procedure was followed using *N*‐Boc‐piperidine‐4‐carboxylic acid **10** (2.04 g, 8.72 mmol), 6‐fluoropyridin‐3‐amine (0.930 g, 8.33 mmol), and 2‐naphthalenecarboxylic acid (0.524 g, 3.12 mmol). Compound **6** was obtained as white solid (0.727 g, 64%); ^1^H‐NMR (400 MHz, DMSO‐*d*_6_): *δ* 10.25 (s, 1H), 8.48–8.38 (m, 1H), 8.16 (ddd, *J* = 8.9, 7.4, 2.8 Hz, 1H), 8.08–7.88 (m, 4H), 7.65–7.45 (m, 3H), 7.21–7.08 (m, 1H), 4.57 (s, 1H), 3.73 (s, 1H), 3.04 (d, *J* = 77.2 Hz, 2H), 2.67 (tt, *J* = 11.4, 3.8 Hz, 1H), 2.07–1.50 (m, 4H); ^19^F NMR (376 MHz, DMSO‐*d*_6_) *δ* −74.59; ^13^C NMR (101 MHz, DMSO‐*d*_6_) *δ* 173.2, 169.1, 159.6, 157.3, 137.7, 134.2, 133.6, 133.1, 132.7, 132.3, 128.3, 127.7, 127.0, 126.7, 126.1, 124.3, 109.5, 46.8, 42.5, 41.0, 28.6, 28.2; HRMS‐ESI (*m/z*): calcd for C_22_H_21_FN_3_O_2_ [M + H]^+^ 378.1618, found: 378.1633.

### 4.9 *N*‐(6‐Fluoropyridin‐3‐yl)‐1‐(quinoline‐2‐carbonyl)piperidine‐4‐carboxamide (**7**)

The above‐mentioned general procedure was followed using *N*‐Boc‐piperidine‐4‐carboxylic acid **10** (2.02 g, 8.73 mmol), 6‐fluoropyridin‐3‐amine (0.930 g, 8.34 mmol), and 2‐quinolinecarboxylic acid (0.527 g, 3.12 mmol). Compound **7** was obtained as white solid (0.470 g, 53%); ^1^H‐NMR (400 MHz, DMSO‐*d*_6_): *δ* 10.26 (s, 1H), 8.50 (d, *J* = 8.4 Hz, 1H), 8.46–8.40 (m, 1H), 8.17 (ddd, *J* = 8.9, 7.4, 2.8 Hz, 1H), 8.04 (dd, *J* = 8.0, 1.4 Hz, 2H), 7.82 (ddd, *J* = 8.3, 6.8, 1.5 Hz, 1H), 7.68 (t, *J* = 8.0 Hz, 2H), 7.14 (dd, *J* = 8.8, 3.2 Hz, 1H), 4.60 (dt, *J* = 13.3, 4.1 Hz, 1H), 3.79 (dt, *J* = 13.6, 4.1 Hz, 1H), 3.17 (td, *J* = 13.5, 12.7, 3.1 Hz, 1H), 2.97 (td, *J* = 12.7, 3.0 Hz, 1H), 2.70 (tt, *J* = 11.3, 3.8 Hz, 1H), 1.98 (dd, *J* = 13.7, 3.6 Hz, 1H), 1.87–1.61 (m, 3H); ^19^F NMR (376 MHz, DMSO‐*d*_6_) *δ* −74.60; ^13^C NMR (101 MHz, DMSO‐*d*_6_) *δ* 173.2, 166.7, 159.6, 157.3, 154.3, 146.1, 137.7, 137.4, 134.2, 132.7, 130.3, 129.0, 128.0, 127.6, 120.2, 109.5, 45.9, 42.5, 40.9, 28.7, 28.0; HRMS‐ESI (*m/z*): calcd for C_21_H_20_FN_4_O_2_ [M + H]^+^ 379.1570, found: 379.1590.

### 4.10 *N*‐(3‐Chlorophenyl)‐1‐(2,3‐dihydrobe‐nzo[*b*][1,4] Dioxine‐6‐carbonyl)piperidine‐4‐carboxamide (**8**)

The above‐mentioned general procedure was followed using *N*‐Boc‐piperidine‐4‐carboxylic acid **10** (1.51 g, 6.54 mmol), 3‐chloroaniline (0.834 g, 6.54 mmol), and 2,3,4*a*,8*a*‐tetrahydrobenzo[*b*][1,4]dioxine‐6‐carboxylic acid (0.529 g, 2.90 mmol). Compound **8** was obtained as a white solid (0.48 g, 53%); ^1^H NMR (400 MHz, DMSO‐*d*_6_) *δ* 10.12 (s, 1H), 7.83 (t, *J* = 2.1 Hz, 1H), 7.51–7.44 (m, 1H), 7.32 (t, *J* = 8.1 Hz, 1H), 7.11–7.08 (m, 1H), 6.95–6.83 (m, 3H), 4.28 (br s, 6H), 3.15–2.74 (m, 2H), 2.65–2.58 (m, 1H), 1.94–1.48 (m, 4H); ^13^C NMR (101 MHz, DMSO‐*d*_6_) *δ* 173.7, 168.9, 144.9, 143.5, 141.2, 133.5, 130.9 (2C), 129.5, 123.3, 120.7 (2C), 119.1 (2C), 118.0, 117.4, 116.5 (2C), 64.7, 43.3, 28.8; HRMS‐ESI (*m/z*): calcd for C_21_H_22_ClN_2_O_4_ [M + H]^+^ 401.1268, found: 401.1287.

### 4.11 1‐(Benzo[*d*][1,3]dioxole‐5‐carbonyl)‐*N*‐(3‐chlorophenyl)piperidine‐4‐carboxamide (**9**)

The above‐mentioned general procedure was followed using *N*‐Boc‐piperidine‐4‐carboxylic acid **10** (1.53 g, 6.54 mmol), 3‐chloroaniline (0.834 g, 6.54 mmol), and 3*a*,7*a*‐dihydrobenzo[*d*][1,3]dioxole‐5‐carboxylic acid (0.488 g, 2.90 mmol). Compound **9** was obtained as a white solid (0.48 g, 55%); ^1^H NMR (400 MHz, DMSO‐*d*_6_) *δ* 10.12 (s, 1H), 7.83 (s, 1H), 7.53–7.22 (m, 2H), 7.17–6.75 (m, 4H), 6.08 (s, 2H), 3.83 (s, 2H), 2.68–2.56 (m, 1H), 2.96 (s, 2H), 1.94–1.45 (m, 4H); ^13^C NMR (101 MHz, DMSO‐*d*_6_) *δ* 173.7, 169.0, 148.6, 147.6, 141.2, 133.5, 130.7 (2C), 130.3, 123.3, 121.6 (2C), 119.1, 117.9, 108.6, 108.0, 101.9, 43.2, 28.8, 23.3; HRMS‐ESI (*m/z*): calcd for C_20_H_20_ClN_2_O_4_ [M + H]^+^ 387.1112, found: 387.1126.

### 4.12 Biology

#### 4.12.1 Determination of Endocannabinoid Hydrolase Activities by a Sensitive Fluorescent Glycerol Assay

Glycerol liberated from 1‐AG hydrolysis was determined with a sensitive fluorescent glycerol assay using lysates of HEK293 cells expressing hABHD6 and hMAGL, as previously described [59]. Briefly, glycerol production, which is coupled via an enzymatic cascade to generate resorufin, was kinetically monitored (0–90 min at room temperature, *λ*_ex_ 530; *λ*_em_ 590 nm) using a Tecan Infinite M200 plate reader (Tecan Group Ltd., Männedorf, Switzerland). Cell lysates (99 μL, 1 μg protein/well) were preincubated for 30 min with the vehicle (DMSO) or the inhibitors (compounds **36** and **2**–**9**) or reference inhibitors (JJKK‐048 as MAGL inhibitor [16] and tetrahydrolipstatin (THL) as ABHD6 inhibitor [59] (1 μL), after which the substrate 1‐AG (12.5 μM final concentration) was added (100 μL). The IC_50_‐values were calculated by Graphpad Prism version 5.03 for Windows, GraphPad Software, San Diego, California USA, www.graphpad.com.

#### 4.12.2 ABPP Using Mouse Whole‐Brain Proteome

Competitive ABPP using mouse whole‐brain proteome was conducted to visualize the selectivity of inhibitors (compounds **36** and **2**–**9**) toward mouse endocannabinoid hydrolases against other serine hydrolases. We used the active site serine‐targeting fluorescent fluorophosphonate probe, carboxytetramethylrhodamine fluorophosphonate (TAMRA‐FP; ActivX Fluorophosphonate Probes (FPs), Thermo Fisher Scientific Inc., Rockford, IL), as previously described [62]. Briefly, brain membranes (50 μg) were first treated for 1 h with DMSO or the selected inhibitors (with appropriate final concentrations ranging from 10 nM to 10 μM) or indicated reference inhibitors at RT, after which TAMRA‐FP labeling was conducted for 1h at RT (final probe concentration 2 μM). The reaction was quenched by adding a 2× gel loading buffer, after which samples with 20 μg protein were loaded per lane, and the proteins were resolved in 10% SDS–PAGE together with molecular weight standards. TAMRA‐FP‐labeled serine hydrolases in samples were analyzed by in‐gel fluorescent gel scanning with the ChemiDoc MP imaging system (BIO‐RAD, Hercules, California, USA) with Cy3 blot application (602/50, Green Epi, Manual Exposure 10–120 s).

#### 4.12.3 Inhibition of Human MAGL‐Mediated Hydrolysis of the Chromogenic Esterase Substrate 4‐NPA

Human MAGL‐mediated hydrolysis of the chromogenic esterase substrate 4‐NPA was measured under conditions designed to closely mimic those used in the glycerol assay in 96‐well plate format. The buffer composition and salt concentrations were identical in both methods. Instead of 1‐AG, 100 μM 4‐NPA was used as a substrate (10 mM stock solution was prepared in DMSO). To ensure adequate solubility of the lipophilic inhibitors, all assays were performed in the presence of 10% DMSO, a concentration previously shown not to interfere with MAGL activity [64]. BSA was omitted from the assay because its intrinsic esterase activity has previously been shown to hydrolyze 4‐NPA. The suitability of 10% DMSO and esterase activity of BSA were also reported in our previous study using 7‐HCA‐substrate in MAGL‐inhibitor testing [64].

HEK293 cell lysates expressing 3HA‐hMAGL used in the glycerol assays were also employed in these experiments. Briefly, cell lysates (99 μL, 5 μg protein/well) were preincubated in clear 96‐well plates at room temperature for 60 min with vehicle (DMSO) or increasing concentrations of inhibitor compound **36** and JJKK‐048 (1 μL), after which 4‐NPA was added (100 μL; final concentration 100 μM). The final DMSO concentration was 10%. Hydrolysis of 4‐NPA was monitored immediately by measuring the absorbance change produced by the reaction product 4‐nitrophenol at 405 nm for 60 min using a Victor Nivo plate reader (Revvity, Turku, Finland). IC_50_ values were calculated using GraphPad Prism for Windows, version 11 (GraphPad Software, San Diego, CA, USA; www.graphpad.com).

#### 4.12.4 Reversibility Assay for MAGL‐inhibitors With Endogenous Substrate 1‐AG

Reversibility of compound **36** and reference compounds (JNJ‐42226314, ATM‐113, and JJKK‐048) in MAGL inhibition were tested with black 96‐well plates suitable for fluorescence measurements, as previously described [18]. Briefly, a dilution‐based protocol in which HEK293 cell lysates expressing 3 HA‐hMAGL (4.5 μL, 5 μg/well protein) were first mixed with inhibitor solutions using micropipette at the indicated concentrations (0.5 μL; DMSO served as vehicle control) followed by 60 min preincubation. Inhibitor concentrations are reported as the concentrations used during the preincubation step. The reaction was subsequently initiated by addition of substrate (25 μM 1‐AG) in assay buffer (volume of 195 μL), resulting in a 40‐fold dilution of the inhibitor (final volume of 200 μL). Kinetic measurements were followed during 90 min incubation with Synergy H1 microplate reader (BioTek Instruments, Winooski, VT, USA) with identical fluorescence parameters (Ex530 nm/Em590 nm) as with the standard glycerol assay for MAGL inhibitors. Note that the inhibition values for studied inhibitors slightly differ from those obtained in the standard assay, as the ratio of inhibitor/MAGL and dilution‐step affect on the result, as previously reported [18, 64]. Inhibitory values were calculated using GraphPad Prism for Windows, version 11 (GraphPad Software, San Diego, CA, USA; www.graphpad.com).

#### 4.12.5 Statistics

Statistical analyses for reversibility results were performed using GraphPad Prism (version 11; GraphPad Software, San Diego, CA, USA). Differences among multiple groups were evaluated using one‐way analysis of variance (ANOVA), followed by Tukey's multiple comparison test. Data are presented as mean ± SEM. Adjusted *p*‐values obtained from Tukey's test were used to determine statistical significance. A *p*‐value ≤ 0.05 was considered statistically significant. Statistical significance is indicated as follows: *p* ≤ 0.05 (*), *p* ≤ 0.01 (**), *p* ≤ 0.001 (***), and *p* ≤ 0.0001 (****).

## Funding

This work was supported by the Academy of Finland (Grant 322724), EDUFI funding (Grant TM‐18‐11003), and Tampereen Tuberkuloosisäätiö.

## Conflicts of Interest

The authors declare no conflicts of interest.

## Data Availability

The data that support the findings of this study are available from the corresponding author upon reasonable request.
